# Primary Immune Regulatory Disorders and Targeted Therapies

**DOI:** 10.4274/tjh.galenos.2021.2020.0724

**Published:** 2021-02-25

**Authors:** Burcu Kolukısa, Safa Barış

**Affiliations:** 1 Marmara University Faculty of Medicine, Division of Pediatric Allergy and Immunology, İstanbul, Turkey; 2 İstanbul Jeffrey Modell Diagnostic and Research Center for Primary Immunodeficiencies, İstanbul, Turkey; 3 The Işıl Berat Barlan Center for Translational Medicine, İstanbul, Turkey

**Keywords:** Primary immune deficiencies, Immune dysregulation, Autoimmunity, Lymphoproliferation, Precision medicine, Targeted therapy

## Abstract

Primary immune regulatory disorders (PIRDs) are a group of diseases belonging to inborn errors of immunity. They usually exhibit lymphoproliferation, autoimmunities, and malignancies, with less susceptibility to recurrent infections. Unlike classical primary immune deficiencies, in autoimmune manifestations, such as cytopenias, enteropathy can be the first symptom of diseases, and they are typically resistant to treatment. Increasing awareness of PIRDs among specialists and a multidisciplinary team approach would provide early diagnosis and treatment that could prevent end-organ damage related to the diseases. In recent years, many PIRDs have been described, and understanding the immunological pathways linked to these disorders provides us an opportunity to use directed therapies for specific molecules, which usually offer better disease control than known classical immunosuppressants. In this review, in light of the most recent literature, we will discuss the common PIRDs and explain their clinical symptoms and recent treatment modalities.

## Abstract (tr)

Primer immündisregülasyon hastalıkları (PİRH), immün sistemin doğuştan gelen bozuklukları içerisinde yer alan bir grup hastalıktır. Çoğunlukla lenfoproliferasyon, otoimmünite ve malignite bulguları sergilerler ve bu grupta tekrarlayan enfeksiyonlara yatkınlık daha az olabilmektedir. Ayrıca diğer klasik primer immün yetmezliklerden farklı olarak otoimmün bulgular (örneğin; sitopeniler, enteropati) hastalığın ilk semptomu olarak çıkabilmekte ve tipik olarak tedaviye direnç gösterebilmektedir. Primer immün disregülasyon hastalıkları hakkındaki farkındalığın artırılması ve multidisipliner yaklaşımlar, erken tanı ve tedaviyi mümkün kılarak, hastalıkla ilişkili gelişebilecek uç-organ hasarlarını önlemeye yardımcı olabilecektir. Son yıllarda, birçok yeni PİRH tanımlanmıştır ve bu hastalıklarla ilişkili immünolojik yolaklarının aydınlatılması bizlere klasik immünosüpresanlar yerine genellikle daha iyi hastalık kontrolü sağlayabilen hedeflenmiş tedavileri kullanma yolunu açmıştır. Bu derlemede, en güncel literatür araştırmaları ışığında, sık rastlanan PİRH’lerin tanımlanması ve bunlarla ilişkili klinik özelliklerin ve yeni tedavi yaklaşımlarının tartışılması hedeflenmiştir.

## Introduction

Recently, many new types of human inborn errors of immunity (IEIs), known previously as primary immunodeficiency disorders, have been discovered with the advance of high-throughput next-generation sequencing technologies. The most recent classification of IEIs by the International Union of Immunological Sciences (IUIS) provides a huge number of single gene defects responsible for unique IEIs, including 430 diseases, and 65 of them have been newly defined in the last two years [1]. Understanding the monogenic causes and underlying mechanisms offers a clearer view of the distinct phenotypes that may be caused by different types of mutations resulting in loss or gain of protein function (LOF/GOF). This also allows for a comprehensive analysis to understand the genotype-phenotype relationship for the related defective genes. 

**Figure 1:**
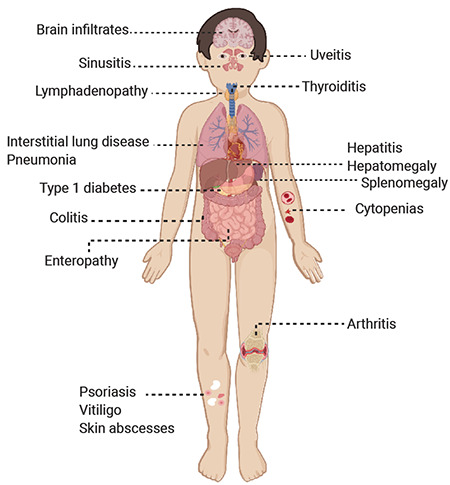
The various immune dysregulatory symptoms observed in patients with inborn errors of immunity.

Primary immune regulatory disorders (PIRDs) are a subgroup of IEIs characterized by heterogeneous clinical phenotypes, predominated by autoimmunity, lymphoproliferation, autoinflammation, and malignancy [2,3,4,5]. These disorders develop from a breakdown in the immune tolerance pathways that can affect different levels of the immune system, modeling various mechanisms for disease development and governing autoimmunity (Table 1). Mutations in various genes have been associated with these diseases and the prototype disorder is immune dysregulation, polyendocrinopathy, enteropathy, X-linked (IPEX) [6,7,8], caused by mutations in the *Forkhead Box P3 (FOXP3)* gene, leading to defective CD4+CD25+ regulatory T-cell (Treg) production and described as a Treg defect (Tregopathies; Table 2) [9]. Additional studies later revealed many different genes that pave the way for reclassification of the patients previously described as having combined immune deficiency (CID) or common variable immunodeficiency (CVID) to monogenic PIRD; some of these cases are also accepted as IPEX- and ALPS-like disorders [2,7]. The presenting symptoms might be quite different than increased frequency of infections and the underlying immune dysregulatory disorders can lead to multiple autoimmunities, lymphoproliferation, and malignancies (Figure 1). A recent study of 2,183 PIRDs reported one or more autoimmunity and inflammatory events in 26.2% of patients and stated that autoimmune disease was a negative prognostic factor for survival in cases of IEIs [10]. Some patient cohorts with refractory cytopenia, such as Evans syndrome, were reported to have a 65% rate of diagnosis with monogenic defects causing IEIs [11]. In fact, many PIRD patients will have a delayed diagnosis of the IEI, since the cardinal symptoms of the disease can be autoimmunity with a less prominent history of infections. Thus, increasing awareness of PIRDs among specialists and a multidisciplinary team approach would provide early diagnosis and treatment that can prevent end-organ damage related to these diseases.

In general, IEI patients are at an increased risk of severe infections and immunosuppressive therapy is required for many of them, with careful consideration of the balance between benefits and harmful side effects. Thus, immune dysregulation makes the treatment more challenging, requiring optimal doses of immunosuppression with careful monitoring of infections. Therefore, in recent years, mechanism-based therapeutic approaches rather than general immunosuppression have been implemented to improve patients’ clinical conditions and prevent infections. This new concept of therapeutic approaches, known as targeted therapies or precision therapy, is a newly discovered area in medicine in which medical treatment is tailored according to the affected pathways of disease. In many PIRDs, precise therapies have altered the aberrant immune response and resolved the severe autoimmune phenomena in patients, providing better disease control compared to conventional immunosuppressants [2,12,13,14]. However, long-term effects and risk of infection and malignancy are ambiguous for these therapies and need further clarity. Some patients with severe phenotypes and partial response to multiple immunosuppressants necessitate more curative therapy like hematopoietic stem cell transplantation (HSCT) or gene therapy.

Herein, we will present the most common clinical features of recently described PIRDs and also discuss the targeted therapies that can be introduced for these specific disorders.

## Lipopolysaccharide-Responsive Beige-Like Anchor Deficiency

**Figure 2:**
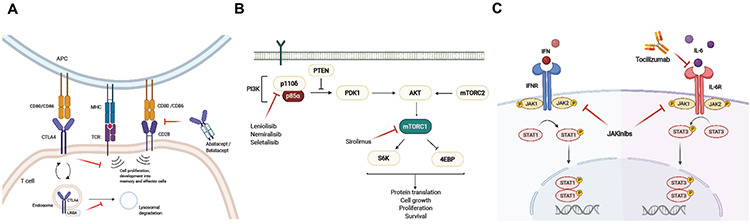
Mechanisms of action of targeted therapies in PIRDs. (A) CTLA4-Fc-fusion proteins (abatacept, belatacept). (B) Inhibitors of the PI3K complex (leniolisib, nemiralisib, seletalisib) and mTOR inhibitor (sirolimus). The PI3K signaling pathway activates AKT serine/threonine kinase, which then phosphorylates mTOR and promotes protein translation, cell growth, survival, and proliferation. (C) JAK inhibitors (jakinibs) and anti-IL-6 (tocilizumab). Created by BioRender.com. PTEN: Phosphatase and tensin homolog; PDK1: phosphoinositide-dependent kinase-1; AKT: AKT serine/threonine kinase; S6K: ribosomal protein S6 kinase; 4EBP1: eukaryotic translation initiation factor 4E-binding protein; mTOR: mechanistic target of rapamycin complex.

Lipopolysaccharide-responsive beige-like anchor (LRBA) deficiency is an autosomal recessive (AR) immune dysregulation disorder that manifests with recurrent sinopulmonary infections, hypogammaglobulinemia, lymphoproliferation, and autoimmunity [15,16,17]. LRBA protein plays a key role in intracellular trafficking of the immune checkpoint inhibitor protein cytotoxic T-lymphocyte antigen-4 (CTLA4) by releasing it from lysosomal degradation and allowing the protein to re-circulate to the cell surface (Figure 2A) [16]. LRBA deficiency results in reduced Tregs with very low CTLA4 expression, which elucidates the phenotypic overlap between LRBA deficiency and CTLA4 haploinsufficiency discussed later in this review [18,19]. As a consequence, patients can present with CVID, ALPS-like, or IPEX-like disease [15,20] with various autoimmunities encompassing hemolytic anemia, thrombocytopenia, enteropathy, arthritis, type 1 diabetes, vitiligo, alopecia, uveitis, and optic neuritis [21,22]. Malignancy (lymphoma and gastric adenocarcinoma) was also reported in some patients. The majority of patients have hypogammaglobulinemia, predominantly as low immunoglobulin (Ig)A and IgG concomitant with poor vaccine responses [15,23]. Immunophenotypic analysis of patients shows reduced CD3+ T-cells, predominantly with memory phenotype, and increased double-negative T-cells with diminished total B-cells accompanied by increased naive (CD27-IgD+) and reduced class-switched memory (CD27+IgD-) cells. Increased activated B-cells (CD21^low^CD38^low^) were also demonstrated compared to healthy controls [15,22]. Inflated frequency of circulating follicular helper T-cells (TF_H_, CD4+PD1+CXCR5+) is the other prominent disease feature and it is found to be associated with impaired CTLA4 signaling [15,18].

Thanks to the discovery of LRBA and related immune pathways, directed therapy has been implemented for many patients, resulting in promising responses in controlling disease symptoms. Impaired Treg cell function with defective activation of mechanistic target of rapamycin 1 and 2 in LRBA deficiency paves the way to use sirolimus as a controller drug to restrain the activity of the disease [24,25]. The identification of CTLA4 as an important immune regulator has also led to the discovery of a fusion protein consisting of the extracellular domain of CTLA4 fused to the Fc region of IgG1 (abatacept and belatacept), acting as a CTLA4 mimetic and helping to restrain the inflammatory manifestations in rheumatoid arthritis [26]. Later studies suggested the effectiveness of abatacept in controlling disease-related immune dysregulatory phenotypes in LRBA deficiency [15,16] and soluble (s) CD25 and cTFH cells were described as beneficial biomarkers to monitor disease activity during abatacept therapy [18]. More recently, we reported a prospective study showing the long-term effect of abatacept in 22 LRBA-deficient patients [15]. The most common clinical features of the patients were recurrent infections (86.4%), immune dysregulation (72.7%), and lymphoproliferation (72.7%). The median duration of abatacept therapy was 12.5 months (range: 5-33 months). Abatacept showed the best complete remission for lymphoproliferation followed by chronic diarrhea and immune dysregulatory symptoms. Interestingly, from the autoimmune perspective, more favorable responses were achieved for hematological autoimmunities (hemolytic anemia and thrombocytopenia), while type 1 diabetes mellitus (DM) was not controlled well with abatacept. The study also demonstrated the efficacy of the dosing intervals used for the patients. Receiving abatacept at 1-week or 2-week intervals provided more disease control compared to the 4-week regimen. Using the cTFH cells as a biomarker for disease control over time revealed a good correlation with disease activity. No serious side effects were reported related to abatacept, expect for newly developed mild eczema in two patients. It was demonstrated in this study that abatacept is safe and provides good disease control. Additionally, using abatacept as a bridge therapy before transplantation can ameliorate patients’ clinical and immunological status and provide better transplantation outcome [15]. Finally, experimental studies have determined that hydroxychloroquine can correct CTLA4 expression in cases of LRBA deficiency. However, the immunomodulatory effect of this drug should be tested in more patients [16]. Patients with severe phenotypes were transplanted with complete remission observed in only 25%, suggesting that the transplantation would have a better outcome when conducted earlier with less disease-related burden [27].

Another recent multicenter study with 76 LRBA-deficient patients evaluated various treatment modalities, including conventional and targeted immunosuppressants and HSCT. The study showed 82.7% survival rate in patients who did not receive transplantation and 70.8% for transplanted patients. Of the total 17 HSCT survivors, 7 were in complete remission and 5 were in good partial remission without treatment (70.6%). In contrast, only 5 of 43 patients without transplantation were free of immunosuppression (11.6%). Although the long-term survival probabilities of transplanted and non-transplanted patients were found to be comparable, immune deficiency and dysregulation activity scores were significantly lower in surviving patients after HSCT compared to patients who had received abatacept or sirolimus. This study’s results also delineated the poor outcomes in the patients who had higher disease burden, longer disease duration before HSCT, and lung involvement. This study addressed the requirement for transplantation at an early stage before disease progression [28].

Overall, the long-term effect of targeted therapy compared to HSCT in LRBA deficiency is still unclear and needs to be addressed with further studies.

## Cytotoxic T-Lymphocyte Antigen-4 Haploinsufficiency

CTLA4 is a crucial inhibitory immune regulator that is responsible for the Treg function of maintaining self-tolerance and downregulating immune response [12,29]. It is constitutively expressed on FOXP3+ Treg cells and is also induced upon activation of conventional T-cells [30]. Competing with the CD28 co-stimulating receptor, CTLA4 attaches the ligands CD80 and CD86 to the surface of antigen-presenting cells, thus inhibiting the stimulatory effect of CD28 on T-cells (Figure 2A) [16,29,31].

Germline mutations in the *CTLA4* gene cause the disease known as CTLA4 haploinsufficiency. Previously, CTLA4 patients were considered as having CVID, with some manifestations of autoimmune cytopenia and lymphocytic infiltration findings in solid organs such as the lungs, the gastrointestinal tract, and the central nervous system. However, after the discovery of autosomal dominant disease, it was accepted as a PIRD [32,33]. Since CTLA4 is an important regulator protein for Tregs, low expression leads to defective suppression function [32]. This defect is the main driver of the immune dysregulation observed in this disease. The disease shows incomplete penetrance with variability in the clinical presentation among individuals carrying the same mutation in one family. In 2018, Schwab et al. [34] described a large cohort of 133 patients with CTLA4 haploinsufficiency, where penetrance was found to be between 60% and 70%. Clinical manifestations include recurrent respiratory tract infections, bronchiectasis, interstitial lung disease, severe enteropathy, life-threatening autoimmune multilineage cytopenias, type 1 diabetes, thyroiditis, arthritis, and alopecia. Immunological findings mainly consist of hypogammaglobulinemia, impaired specific antibody responses, reduced CD4+ T-cells with the predominance of decrease in naive and increase in memory CD4+ T-cell compartment, and defective B-cell maturation characterized by increased naive B-cells and progressive loss of memory with increased CD21low B-cells.

As denoted previously, due to the defective Treg function, overactivation of the T-cells results in lymphoid organ infiltrates [33]. Thus, patients with CTLA4 deficiency have been treated with selective mTOR inhibitors like sirolimus, which specially inhibit effector T-cells and eventually succeed in controlling the autoimmunity [33,34]. Schwab et al. [34] summarized the results of 13 patients with rapamycin, showing that 61.5% of them had good responses for lymphoproliferation, cytomegalovirus (CMV) infection control, enteropathy, and erythrocyte transfusion dependence in patients with pure red cell aplasia. In particular, patients should be monitored for common side effects related to rapamycin during follow-up (e.g., oral ulceration, hyperlipidemia, decreased renal function, myelosuppression).

It was shown that abatacept and belatacept were effective targeted treatments to control immune dysregulation and related conditions in CTLA4 deficiency. Abatacept, a medication previously approved by the FDA for rheumatoid arthritis, has demonstrated powerful clinical response in CTLA4-deficient patients [34]. In the cohort of Schwab et al. [34], 11 of 14 patients had resolved symptoms related to enteropathy, granulomatous-lymphocytic interstitial lung disease, thrombocytopenia, and lymphoproliferation and some improvement of optic neuritis. Of note, severe side effects can be observed with treatment, such as viral infection re-activation [Epstein-Barr virus (EBV)], recurrent respiratory tract infections, and agranulocytosis, and physicians should be mindful of these side effects during drug maintenance. Long-term efficacy and other side effects of abatacept, like immunosuppression-associated infections or malignancies, are still elusive in CTLA4 deficiency and need more investigation.

## Activated PI3K Deficiency Syndrome (APDS)

The phosphoinositide 3-kinase (PI3K) signaling pathway is located downstream of the surface membrane of cells and is required for the maintenance of basic cellular biology, including growth, differentiation, proliferation, motility, and survival (Figure 2B) [35]. Molecular defects related to PI3K signaling were recently discovered and described as activated PI3K deficiency syndrome (APDS). There are two forms of the disease depending on the defective molecular basis of the PI3K enzyme. APDS-1 is caused by heterozygous GOF mutations in *PIK3CD* and APDS-2 is caused by heterozygous LOF mutations in *PIK3R1*, and both disorders result in hyperactivation of the PI3K pathway [36]. APDS is classified as CVID according to the recent IUIS classification [1]. However, patients were followed with different clinical diagnoses such as CID, hyper-IgM syndrome, or antibody deficiency before genetic analysis. The disease presents with susceptibility to severe bacterial *(Streptococcus pneumoniae, Haemophilus influenzae*) and viral infections, especially CMV and EBV, and growth retardation and colitis. Important clinical aspects include generalized lymphoproliferation (hepatosplenomegaly, lymphadenomegaly, nodular mucosal lymphoid hyperplasia), growth retardation, increased risk of lymphoid malignancies and solid organ tumors, increased autoimmunity (mainly hematological-hemolytic anemia and immune thrombocytopenia), and inflammation [36,37].

The first published cases of APDS were described concurrently by two different groups [38,39], showing that mutations of the *PIK3CD* gene, encoding the catalytic p110δ subunit of the PI3Kδ enzyme, resulted in elevated lipid kinase activity of PI3Kδ and led to hyperactivation of PIP3-AKT-mTOR-S6K signaling (Figure 2B). Shortly after this discovery, heterozygous splice-site mutations in *PIK3R1*, encoding regulatory p50a, p55a, and P85a subunits of the PI3K enzyme, were found to be the other underlying causes of APDS [40,41]. Patients with APDS present with overlapping clinical features related to immunodeficiency and immune dysregulation. The main features of the disease are characterized by recurrent respiratory tract infections, bronchiectasis, herpesvirus infections, autoimmunity, non-neoplastic lymphoproliferation, lymphoma, neurodevelopment delay, and growth retardation [42,43]. Interestingly, some of these features are more common in one form of disease, such as APDS-1 showing a higher rate of bronchiectasis and lower rate of lymphoma when compared to APDS-2. As noted, growth retardation was only described in patients with APDS-2 [36].

The laboratory findings of APDS include decreased proportion of naive T-cells and increased effector memory T-cells predominantly with exhausted CD8+ T-cells [36]. There are also elevated TF_H_ cells in some cases. The class-switch recombination of B-cells is impaired, and patients have reduced numbers of switched memory B-cells and increased transitional B-cells as well as variable degrees of hypogammaglobulinemia with poor response to vaccinations. Some patients present with a hyper-IgM phenotype, with low IgG and IgA levels while IgM is normal or high [13].

As is the case for many other primary immunodeficiencies, conventional treatment of APDS includes immunoglobulin replacement therapy and antimicrobial prophylaxis [44]. Autoimmune cytopenias may be responsive to corticosteroids, rituximab, and/or splenectomy [42]. Functional tests demonstrated augmented AKT and S6 phosphorylation in T- and B-cells as a result of heightened mTOR signaling in APDS patients [39]. Accordingly, the use of mTOR inhibitors was observed as a reliable therapy for patients. Until recently, anti-CD20 monoclonal antibody and mTOR inhibitors (rapamycin) were administered to control the lymphoproliferation, which showed variable responses [44]. A multicenter study conducted with the ESID-APDS registry including 26 patients (17 with APDS-1 and 9 with APDS-2) demonstrated the maximum effect of sirolimus on non-malignant lymphoproliferation with 32% and 44% of patients showing complete and partial responses to sirolimus, respectively [45]. However, enteropathy and cytopenias were less controlled (absence of response in 60% and 69%, respectively) [36].

HSCT has been performed to treat life-threatening infections, lymphoproliferation with insufficient control despite therapies, and lymphomas [36,42,43,46,47]. The overall survival after transplantation was 81% and 78% in the studies of Nademi et al. [47] and Okano et al. [46], respectively. Lower survival rates can be attributed to severe phenotypes complicated with end-organ damage in the majority of patients and the high rate of posttransplantation complications. Better defined transplantation cohorts are required to determine the place of HSCT in APDS.

The discovery of the mechanism of APDS brings the opportunity of using selective PI3Kδ inhibitors as a targeted therapy [48,49]. There are ongoing clinical trials with the oral p110δ subunit inhibitor leniolisib (NCT02435173, NCT02859727) and inhaled nemiralisib (NCT02593539). Oral administration of leniolisib in six APDS-1 patients over 12 weeks reduced lymphoproliferation, controlled cytopenias, and decreased the senescent T- and naive B-cells [49]. There is no information regarding control of symptoms associated with respiratory or gastrointestinal symptoms. No significant side effects were reported, but currently the long-term efficacy is being evaluated with an extension study (NCT02859727). Nemiralisib is an inhaled PI3Kδ inhibitor, currently being investigated as an antiinflammatory agent for the treatment of chronic obstructive pulmonary disease [50]. It is an agent intended for APDS patients with lymphoproliferation and respiratory disease in hopes of preventing bronchiectasis development or its worsening [36]. There is also a third drug, seletalisib, a potent PI3Kδ inhibitor, evaluated in a phase 1b and extension study and found to have improved peripheral lymphadenopathy, lung function, thrombocytopenia, and enteropathy in the patients [51].

## Signal Transducer and Activator of Transcription 1 Gain-of-Function Disease

Signal transducer and activator of transcription 1 (*STAT1*) plays a crucial role in the signaling of many cytokines for innate and adaptive immune responses to viruses and intracellular bacteria [52,53]. STAT1 conducts the responses of many cytokines [interferon (IFN)-α/β/λ, IFN-γ, interleukin (IL)-2, IL-6, IL-21, IL-27] via JAK1, JAK2, and JAK3 to the nucleus and thus facilitates immune and inflammatory processes that are important for cellular biology [53,54].

The *STAT1* gene is the target of heritable LOF or GOF mutations that give rise to distinct clinical phenotypes [55]. While AD *STAT1* LOF mutated patients suffer from infections with mycobacteria and other macrophage-bound bacteria but do not demonstrate undue susceptibility to viral infections, AR hypomorphic *STAT1* LOF mutated patients are prone to both mycobacterial and viral infections [52,56,57]. AD GOF *STAT1* mutations are frequently associated with chronic mucocutaneous candidiasis (CMC), immunodeficiency, and autoimmune phenomena, accompanied by increased expression of IFN-stimulated genes (ISGs) and reduced T-helper cell type 17 (TH17) responses [58]. Although the ISG upregulation in these patients has been speculated to cause autoimmune manifestations, the precise mechanisms leading to autoimmunity are largely unknown. AD-GOF mutations in *STAT1* result in increased STAT1 phosphorylation or delayed/impaired dephosphorylation after activation with cytokines [52,54].

In a cohort reported by Toubiana et al. [59] consisting of 274 subjects, 98% of them were described to suffer from CMC, with median age at onset of 1 year. Bacterial (74%), viral (38%), and invasive fungal (10%) infections were reported. The most common isolated microorganisms were *Candida albicans* (82%), *Staphylococcus aureus* (36%), *Mycobacterium tuberculosis *(35%), and herpes simplex virus (27%). Susceptibility to fungal infections was not limited to *Candida* or *Aspergillosis*; *Mucormycosis*, *Coccidioidomycosis*, and *Histoplasmosis* were also reported among the patients [58,60]. Some patients suffered from life-threating viral infections like CMV and EBV [52,61]. An immune dysregulation component was noted in 37% of patients, including hypothyroidism, type 1 diabetes, cytopenias, systemic lupus erythematosus, enteropathy, arthritis, and multiple sclerosis. Aneurysm occurred at a higher rate among the patients (6%), usually located in the cerebral vascular system. The plausible mechanism for aneurism susceptibility could be mycotic translocation, since *Candida* hyphae were identified in some of the microbiological examinations of aneurysms [62].

The degree of immune deficiency described among the patients is variable, and most of them show normal peripheral blood lymphocyte distributions [59]. However, the abnormal immune defects are mainly related to low memory B-cells. Some patients present with decreased proportions of T-, B-, and/or NK-cells and hypogammaglobulinemia [52,59]. Interestingly, NK-cell abnormalities with more immature phenotype and low perforin expression were also described in this disease, contributing to the susceptibility to viral infection [63].

Medical treatment is mainly based on long-term antifungal prophylaxis, which may be complicated when azole-resistant strains develop. Antibacterial and antiviral prophylaxis may also be needed, as well as immunosuppressive drugs for immune dysregulation [55,59]. However, to date, the treatment modalities for patients are not well established and further patient descriptions with long-term treatment outcomes are needed.

Oral therapy with jakinibs (JAK inhibitors) inhibits Janus kinase (JAK) activation and attenuates cytokine receptor-mediated STAT1 phosphorylation, thereby controlling the inflammatory processes of the disease (Figure 2C). While ruxolitinib and baricitinib mainly inhibit JAK1 and JAK2, tofacitinib blocks JAK1 and JAK3 activation. However, the response rate of patients to the targeted therapy reportedly ranges from favorable disease control to worsening of fungal infections as a complication of treatment [54,64,65]. It was shown that ruxolitinib provided complete remission in CMC and autoimmune phenomena (e.g., alopecia areata) for *STAT1* GOF patients [66]. In vitro studies have also shown that ruxolitinib significantly normalizes the exaggerated cytokine-induced response of STAT1 [54,67]. A small cohort consisting of 11 *STAT1* GOF patients with multiple autoimmune complications treated with ruxolitinib demonstrated favorable clinical outcomes [67]. Five patients had CMC, one had disseminated coccidioidomycosis, six had autoimmune cytopenias or autoimmune hepatitis, and five had autoimmune enteropathy that led to failure to thrive. Ninety percent of patients (10/11) receiving ruxolitinib in this cohort had substantial improvement in their immune dysregulatory manifestations and CMC. Although Forbes et al. [67] thus showed the favorable effect of JAK inhibitors, another report revealed the worsening of fungal infections as a complication of treatment [65]. In *STAT1* GOF disease the regular dose of ruxolitinib was adopted from previous studies, which is usually described as 15-50 mg/m2/dose twice daily [68].

The outcome of HSCT in *STAT1* GOF patients is known to be curative but there are serious posttransplant complications, including graft failure, graft-versus-host disease (GVHD), and bleeding [69]. Recently, Kiykim et al. [70] reported two patients with *STAT1* GOF mutations treated with HSCT due to the severe course of the disease. One of the patients had refractory oral candidiasis unresponsive to oral fluconazole therapy, CMV pneumonitis, mycobacterial lung disease, and autoimmune hepatitis, while the other patient had severe lung infections, recurrent CMV viremia, and resistant oral candidiasis. Both patients received HSCT from HLA-matched donors and the HSCT was successful in one, showing sustained complete disease remission. However, the other patient experienced secondary graft failure and died due to CMV pneumonia and pulmonary hemorrhage. Hypothetically, an exaggerated IFN-γ response can be associated with poor engraftment and a negative outcome after transplantation in these patients. Therefore, pretransplant use of ruxolitinib to suppress hyperactive IFN responses may allow better immune reconstitution by controlling the hyperinflammatory process and reducing the posttransplant complications as shown for patients with hemophagocytic lymphohistiocytosis (HLH) [71].

Our experience suggests that ruxolitinib therapy can offer better disease management before transplantation, which may be beneficial in enhancing the survival rate of patients after HSCT. The treatment dose for ruxolitinib can be adjusted in the patients based on the response normalization observed in functional assays. However, further studies are needed to explore the most effective dose and adverse effects of ruxolitinib treatment in this disease.

## Signal Transducer and Activator of Transcription 3 Gain-of-Function Disease

*STAT3* controls intracellular signaling of various cytokines and growth factors, playing a role in T_H_17 activation while restricting the development of Tregs [72]. The first patients with heterozygous *STAT3* GOF mutations described in 2014 by Flanagan et al. [73] had infantile diabetes and other early-onset autoimmune disorders, including juvenile-onset arthritis, type 1 diabetes, enteropathy, and thyroiditis. Other reports broadened the clinical phenotype to lymphoproliferation as hepatosplenomegaly and lymphadenopathies, short stature, recurrent infections with viral and fungal agents, non-tuberculous mycobacteria, and interstitial pneumonia. Autoimmune manifestations of the disease are multi-systemic; DM, hypothyroidism, cytopenias, enteropathy, hepatitis, and inflammatory lung disease are reported [74,75,76]. Two patients also developed malignancies with Hodgkin’s lymphoma in one case and large granulocytic leukemia (LGL) in the other, as shown in somatic *STAT3* GOF mutations in 30%-40% of LGL patients [72]. Evaluation of family members with genetic mutations revealed a mild phenotype of the disease, delineating the possible incomplete penetrance and variable expressivity of *STAT3* GOF mutations [75]. Activation in *STAT3* may lead to autoimmunity by damaging the development of regulatory T-cells, presumably because of enhanced IL-6 signaling, and stimulating the expansion and activation of T_H_17 cells [77]. However, T_H_17 cell expansion has not been shown in the majority of *STAT3* GOF patients, suggesting other possible mechanisms related to autoimmunity [76].

Recently, Fabre et al. [75] reviewed 42 *STAT3* GOF patients and investigated the immunological aberrations in more detail. The immunological characteristics of the disease include T-cell lymphopenia, increased percentage of double-negative TCRαβ-positive T-cells, defects in B-cell maturation accompanied by hypogammaglobulinemia, and reduced numbers of natural killer cells. Low Treg cell frequencies were observed in most reported patients. The T_H_17 cells levels were studied in four patients but were found to be high in only one patient, while they were low in the others [76].

Several immunosuppressive drugs have been administered in this disease to control the immune dysregulatory symptoms, resulting in variable responses [75]. On the other hand, targeted treatment directed to inhibit IL-6 signaling was shown to be effective for controlling the disease activity [74]. In 2015, Milner et al. [74] reported a 10-year-old patient with scleroderma, arthritis, autoimmune hemolytic anemia, and autoimmune hepatitis treated with the IL-6 receptor inhibitor tocilizumab (Figure 2C). The arthritis and scleroderma of the patient were improved dramatically over 1 year of treatment. Furthermore, other autoimmune features such as hepatitis, interstitial lung disease, enteropathy, and lymphoproliferation were also alleviated. In a study with six *STAT3* GOF patients, treatment with jakinibs was reported with good control of disease activity [67]. All patients had severe autoimmunity including enteropathy, hepatitis, cytopenias, lymphoproliferation, serositis, and severe growth failure and previously received broad immunosuppressive drugs (e.g., corticosteroids, methotrexate, rituximab, mycophenolate mofetil, cyclophosphamide) and were still receiving concomitant immunosuppressive medications at the time of jakinib application (ruxolitinib in most, tofacitinib in one patient). Additionally, tocilizumab, a human IL-6 receptor-blocking monoclonal antibody, was used as an adjunctive treatment in all six patients. Three of the patients received tocilizumab prior to, two simultaneously, and one after treatment with jakinibs. The results suggested that the combination of a jakinib and IL-6 receptor blockade is an effective strategy in resolving immune dysregulation in difficult cases of *STAT3* GOF disease.

Finally, treatment with HSCT was reported in five cases of *STAT3* GOF. Remission of autoimmunity and immune constitution was achieved in only one of these patients, while the other four patients died from various complications such as GVHD and infections [75]. Further reports with well-defined transplantation regiments, including therapy targeted to the *STAT3* pathway, are needed to better understand the efficacy of HSCT in controlling the disease activity.

## Hemophagocytic Lymphohistiocytosis

HLH [78] is a severe hyperinflammation disorder caused by extreme macrophage and cytotoxic lymphocyte activation. It was first described in 1952 in two siblings suffering from cytopenias, coagulopathy, and persistent fever [79]. Since then, associated clinical features and diagnostic criteria have been established and further broadened [80]. Different clinical presentations include hepatitis, acute liver failure, and neurological findings such as altered mental state or seizures. Diagnostic laboratory findings typically show elevated ferritin levels, triglyceride, and sCD25, as well as low levels of fibrinogen. IL-18 is highly elevated in some HLH patients, particularly in cases of XIAP deficiency, patients with *NLRC4* mutation, and macrophage activation syndrome (MAS) because of systemic juvenile idiopathic arthritis (sJIA).

Several monogenic defects that primarily manifest with HLH development, including *PRF1*, *UNC13D*, *STX11*, *STXBP2*, *RAB27A, LYST*, and *AP3B1*, are considered as “primary HLH.” These patients need HSCT for complete disease cure. Other genetic causes of HLH include primary immunodeficiencies such as XIAP and SAP deficiencies or *MAGT1* and *ITK* mutations giving rise to a susceptibility to EBV infection and EBV-related HLH. Secondary HLH might be caused by infections, malignancy, metabolic diseases, or other primary immunodeficiencies. Distinguishing between primary and secondary HLH is important to better understand the disorder and manage treatment [81,82]. The main treatment regimen for HLH is the combination of high-dose dexamethasone and etoposide [83], published as a prospective trial by the Histiocyte Society in 1994 (HLH-1994) [84]. Cyclosporine was often used as a maintenance drug. However, complete response to this treatment was only seen at a rate of 53%, and additional or alternative treatments were needed for non-responders and those who experienced a relapse of HLH after initial treatment.

Recently, different targeted therapies are being considered as treatment options. These targeted biologic agents usually used in refractory HLH are anakinra, alemtuzumab, and emapalumab. Anakinra, an IL-1 receptor antagonist, was used by Behrens et al. [85] for a 14-year-old patient with cytophagic histiocytic panniculitis and secondary HLH after initial therapy with methylprednisolone, etoposide, and cyclosporine. The mental status, cytopenia, and other laboratory parameters improved within 2 days of therapy and organomegaly resolved after 1 week of anakinra. Another report on the use of anti-IL-1 treatment in two patients with HLH/MAS (one with a diagnosis of Kawasaki disease and the other sJIA) was published by Miettunen et al. [86]. These patients were also treated with methylprednisolone, etoposide, and cyclosporine and both were reported to have achieved complete resolution of HLH after the 10th day of initiation of anakinra.

Alemtuzumab, a monoclonal antibody against the CD52 antigen, acts by depleting CD52-expressing cells such as T- and B-cells, NK-cells, monocytes, and macrophages. It is used both in refractory HLH as a second-line agent and as part of reduced-intensity conditioning regimens in HSCT. In 2013, Marsh et al. [87] reported a cohort of 22 pediatric and young adult patients with HLH treated with alemtuzumab. Although complete response was not achieved in any of the patients, 64% had an overall partial response and 77% of the patients survived to undergo HSCT. There were few adverse events; four patients had fever, one patient had urticaria, four patients had transient worsening of neutropenia, and two patients had transient worsening of thrombocytopenia. CMV and adenovirus viremia were observed in some patients.

Another targeted therapy directed at B-cells is rituximab, usually used to control EBV-related HLH [88]. In a retrospective study involving 42 patients with EBV-related HLH who received an average of three rituximab infusions at a median dose of 375 mg/m^2^, 43% of patients were observed to have improved clinical status [78].

A human IgG1 monoclonal antibody against IFN-γ, emapalumab, received approval for primary HLH in both pediatric and adult patients in 2018 [89]. It binds to both soluble IFN-γ and IFN-γ bound to receptors with high affinity. A clinical trial (NCT01818492) assessed the efficacy and safety of the drug in pediatric patients, including 34 patients with primary HLH who had previously received conventional therapy with poor outcomes. The median age of the patients was 1 year and secondary HLH patients were excluded from the study. All participants received emapalumab at a dose of 1 mg/kg every 3-4 days, concomitant to dexamethasone at 5 or 10 mg/m^2^/day. Emapalumab dosing was increased by 10 mg/kg according to the patients’ laboratory responses. The treatment duration was up to 8 weeks or extended until HSCT. The overall response rate to the drug was 64.7%. However, only 26% of the patients achieved complete response, while the remaining patients showed partial response. Infections were a common adverse event (56%) during the use of this drug. Monitoring of viral and mycobacterial infections (EBV, CMV, adenovirus, tuberculosis) and prophylaxis for herpes zoster and *P. jirovecii *are recommended during therapy with anti-IFN-γ [90].

Considering that cytokine targeting in HLH is important, recently the JAK1/2 inhibitor ruxolitinib has been shown effective in mouse models of HLH, inhibiting IFN-γ, IL-6, and IL-12 secretion and improving clinical symptoms [91]. In 2017, Broglie et al. [92] reported an 11-year-old male patient treated with ruxolitinib for refractory HLH. The patient had clinical improvement of fever, respiratory failure, and liver functions within 24 hours. Recently, Wang et al. [93] published a study including 34 patients aged 2-70 years with refractory/relapsed HLH. They reported that 14.7% of the patients (5 of 34) achieved complete response and 58.8% (20 of 34) achieved partial response. Patients who had a response to ruxolitinib had decreased levels of IFN-γ, IL-18, macrophage inflammatory protein (MIP)-1a, and IFN-γ-IP-10 within 2 to 4 weeks of treatment.

An anti-IL-18 recombinant human antibody (tadekinig alfa) is considered as an option for patients with *NLRC4* mutations or XIAP deficiency who have high IL-18 levels and inflammasome activation. Clinical trial NCT03113760 is still recruiting patients.

## Conclusion

In patients with autoimmunities, especially refractory cytopenias, and lymphoproliferation, PIRDs should be considered for early diagnosis. A multidisciplinary team approach using directed therapies will provide better disease control and outcomes by achieving the best restoration opportunity for the immune system. Long-term data regarding targeted therapy are needed to understand the full efficacy of these drugs in controlling the disease symptoms compared to definitive therapies like HSCT or gene therapy.

## Figures and Tables

**Table 1:**
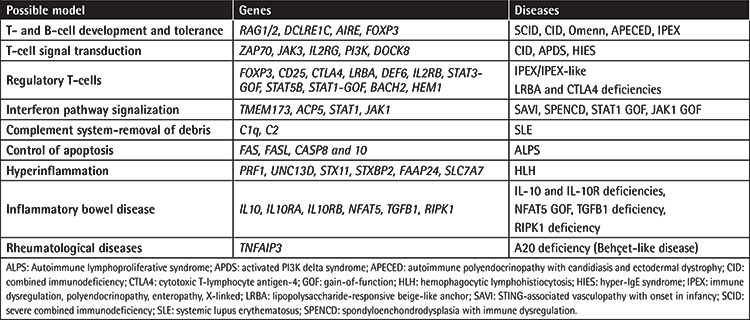
The possible mechanisms for immune dysregulation in PIRD patients.

**Table 2:**
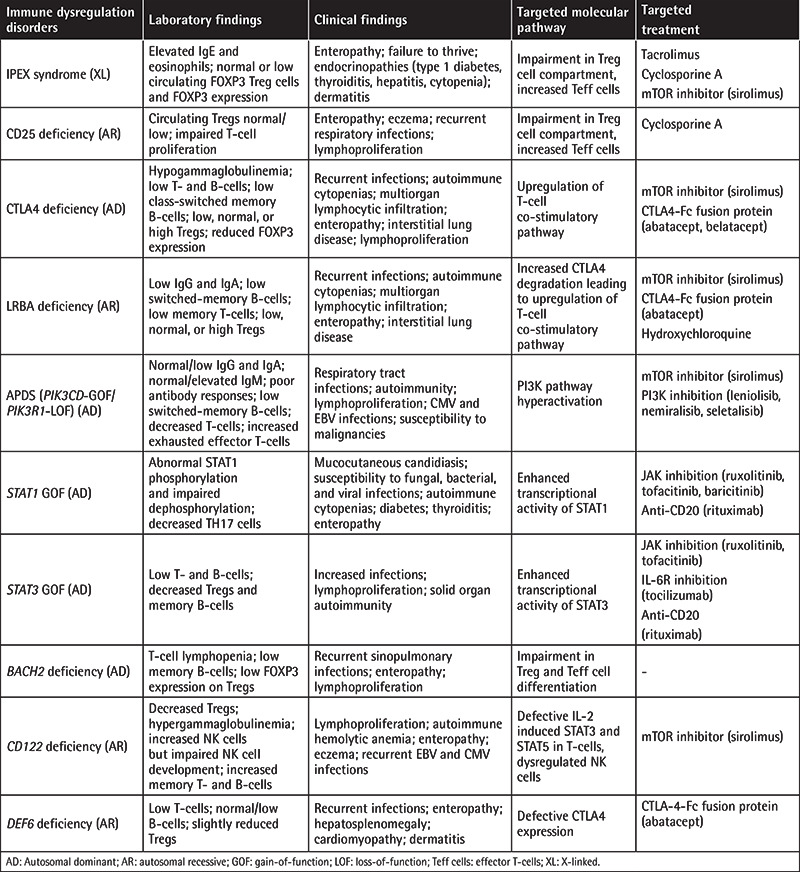
Tregopathies and their main clinical and laboratory features.
